# Association between demographic and radiographic characteristics of the schneiderian membrane and periapical and periodontal diseases using cone-beam computed tomography scanning: A retrospective study

**DOI:** 10.15171/joddd.2017.031

**Published:** 2017-09-20

**Authors:** Azin Khorramdel, Adileh Shirmohammadi, Alireza Sadighi, Masoumeh Faramarzi, Amir Reza Babaloo, Mehrnoosh Sadighi Shamami, Amin Mousavi, Zia Ebrahim Adhami

**Affiliations:** ^1^Department of Periodontics, Faculty of Dentistry, Tabriz University of Medical Sciences, Tabriz, Iran; ^2^Department of Oral and Maxillofacial Surgery, Faculty of Dentistry, Khorasgan Islamic Azad University, Isfahan, Iran; ^3^Department of Prosthodontics, Faculty of Dentistry, Tabriz University of Medical Sciences, Tabriz, Iran; ^4^Department of Pedodontics, Faculty of Dentistry, Tabriz University of Medical Sciences, Tabriz, Iran

**Keywords:** Cone-beam computed tomography, schneiderian membrane, periapical abscess, periodontitis

## Abstract

***Background.*** This study was undertaken to assess the pathological and spatial associations between periapical and periodontal diseases of the maxillary first molars and thickening of maxillary sinus mucosa with cone-beam computed tomography.

***
Methods.*** A total of 132 CBCT images of subjects 20‒60 years of age were evaluated retrospectively. The patients' sex and age and demographic and pathologic findings of the maxillary sinus in the first molar area were recorded, graded and analyzed.

***
Results.*** Approximately 59% of patients were male and 41% were female, with no significant difference in the thickness of schneiderian membrane between males and females. Based on the periapical index scoring, the highest frequency was detected in group 1. Based on the results of ANOVA, there were no significant differences in the frequencies of endodontic‒periodontal lesions and an increase in schneiderian membrane thickness. There were significant relationships between periapical and periodontal infections (P<0.001) and schneiderian membrane thickness. Furthermore, a significant relationship was detected between the thickness of the schneiderian membrane and the distance between the sinus floor and the root apices (P=0.38).

***
Conclusion.*** A retrospective inspection of CBCT imaging revealed that periapical lesions and periodontal infections in the posterior area of the maxilla were associated with thickening of the schneiderian membrane. In addition, there was a significant relationship between the location of maxillary posterior teeth, i.e. the thickness of bone from the root apex to the maxillary sinus floor, and schneiderian membrane thickness.

## Introduction

It is absolutely necessary to replace the lost teeth during dental treatments to restore mastication, speech and esthetics. This is achieved with the use of fixed and removable prosthetic appliances and root-form implants. Currently, use of root-form implants is the best treatment modality to replace the lost teeth, in which the implant is placed within the jaw bones and achieves the required stability through osseointegration between bone and implant.^1,2^

Currently, one of the challenges in dentistry is to place implants in the posterior maxilla in cases in which the density and height of bone are inadequate.^3,4^ Sinus lifting procedures are used to solve such problems.^5^ Radiographic findings make great contributions to treatment planning for sinus lift procedures by revealing the presence of bony septa within the sinus,^6^ thickening of the schneiderian membrane, destruction due to a previous sinus lifting procedure and the presence of pathologic entities within the maxillary sinus, including acute rhinosinusitis or neoplastic processes.^7^

Based on previous studies, 10‒12% of cases of maxillary sinusitis are due to odontogenic infections as a result of the proximity between the roots of maxillary posterior teeth and the maxillary sinus in the posterior maxilla.^8,9^ A study showed that 98% of 135 cases of maxillary sinusitis cases were associated with teeth that had caused changes in the sinus floor integrity.^10^

Apical periodontitis,^11^ periodontal diseases,^11,12^ implant treatment^13^ and tooth extraction^14^ are thought to increase the odds of maxillary sinusitis. Asymptomatic individuals might exhibit a minor increase in maxillary sinus membrane thickness, which is considered normal;^15^ however, if the thickness is >2 mm in MRI examination, it is considered a sign of sinusitis and possible pathologic entities in the sinus.^16^

It is difficult to visualize important maxillary sinus anatomic areas adjacent to the roots of molars due to the superimposition of the adjacent structures.^17-19^ CT scan examinations are usually used for the evaluation of paranasal sinuses.^20,21^

The CBCT technique is a novel 3D imaging modality which has been used for dentomaxillofacial evaluations since 1998. It requires less radiation exposure and yields image quality comparable to that of the CT technique.^12,22-24^ Currently, this technique is used by dentists and otolaryngologists for the evaluation of paranasal sinuses.^17^

The aim of the present study was to use the CBCT technique to evaluate the demographic and radiographic characteristics of the schneiderian membrane and their relationship with periodontal and periapical diseases.

## Methods

In the present retrospective study, the CBCT images of patients, referring to a private oral and maxillofacial radiology center (Kaviani & Johari Oral and Maxillofacial Radiology Center, Tabriz, Iran) from 2013 to 2015, were used. The exclusion criteria consisted of CBCT images of completely edentulous subjects in the maxilla, patients with implants in the maxilla, patients with a history of an acute trauma to the maxillary sinus, patients with suspected cysts in the posterior maxilla and patients with a history of bone grafts in the posterior maxilla.^8^ The inclusion criterion was patients with maxillary first molars. A total of 132 CBCT images of subjects 20‒60 years of age were evaluated, consisting of 78 males and 54 females. The CBCT unit was 3D Accuitomo XYZ Tomography (Morita, Kyoto, Japan) with a voxel size of 0.08 mm. In addition, the mA and kVp were adjusted at 5‒7 and 80, respectively. In all the images, FOV was confined to the maxillary bone in order to produce high-quality images. The images were evaluated using 0.5-mm cross-sections. The images were viewed in a dimly-lit room on a Dell monitor (One Volume Viewer, J Morita, Japan) with a resolution of 1920×1200, using volume-rendering software program (Dell Computer Corp., Ran Drake, TX). All the images were evaluated by two periodontists and one radiologist twice with a one-week interval. In cases in which there was a difference of >0.2 mm between the observers, the images were evaluated again and the mean of the values reported by the observers was reported and used.

**Figure 1:**
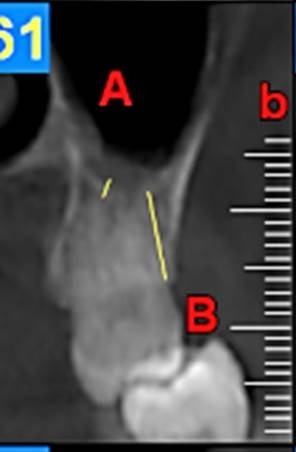
A, the distance between the maxillary sinus floor and the nearest apex of the root of the first molar. B, the distance between the nearest alveolar crest of the first molar on the buccal or lingual aspect.

**Figure 2:**
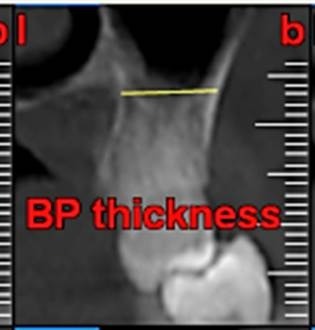
the buccopalatal thickness in the area superior to the apex of the first molar

**Figure 3:**
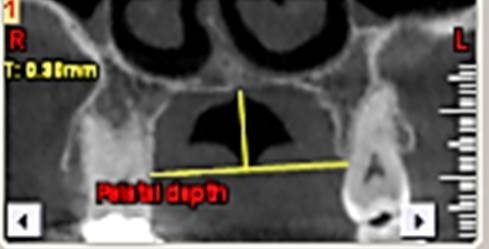
the palatal depth

**Figure 4:**
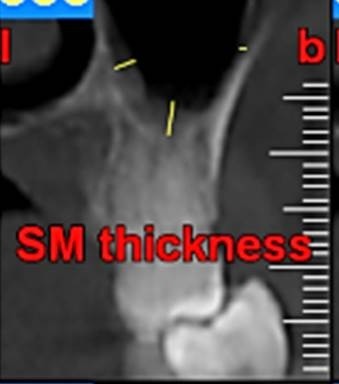
the thickness of the schneiderian membrane

First, the radiographs were evaluated in relation to the endodontic and periodontal problems of the teeth and divided into 5 groups based on periapical index scoring, the periapical status was graded as follows: 1) normal periapical structures; 2) minor changes in bone structure; 3) some changes in bone structure with some loss of minerals; 4) periodontitis with a well-defined radiolucent area; and 5) severe periodontitis with exacerbating features.^25^ The patients’ genders were also recorded. In the next stage, the teeth were evaluated for the presence of periapical and periodontal lesions, followed by determination of the distance between the maxillary sinus floor and the nearest apex of the root of the first molar (Figure 1, A) and the nearest alveolar crest of the first molar on the buccal or lingual aspect (Figure 1, B). Furthermore, the buccopalatal thickness in the area superior to the apex of the first molar was determined in the coronal dimension (Figure 2). In the next stage, a line was drawn perpendicular to the line connecting the palatal crests of the first molars on both sides in order to measure the depth of the palate (Figure 3). Finally, the thickness of the schneiderian membrane was determined at three regions of the maxilla: medial, lateral and inferior. Then the mean of the 3 values was used as a reference (Figure 4).

SPSS 22 was used for the analysis of data, with Pearson’s correlation coefficient, Tukey test and regression analysis. Intra-group differences between the parameters of the present study were analyzed with Tukey test and ANOVA. In addition, Pearson’s correlation coefficient was used to analyze the relationship between the two variables. Furthermore, regression analysis was used to analyze the relationships between the variables.

## Results

Of 132 samples in the present study, there were 78 (59%) in group 1 (male) and 54 (41%) in group 2 (female). T-test was used to analyze independent samples and their distributions.

Concerning endodontic‒periodontal lesions, the highest mean frequency was recorded in group 1 (2.87±2.91) and the lowest was recorded in group 5 (5.70±4.05) (Table 1).

**Table 1:** Periapical index scoring

					95% Confidence Interval for Mean	Minimum		
	N	Mean	Std. Deviation	Std. Error	Lower Bound	Upper Bound	Maximum	
1	100	2.8762	2.91084	.29108	2.2986	3.4538	.64	16.82
2	7	3.8700	3.75068	1.41762	.4012	7.3388	1.29	12.09
3	15	3.1013	2.85271	.73657	1.5216	4.6811	.66	11.04
4	7	6.3243	5.91564	2.23590	.8532	11.7953	2.26	18.59
5	3	5.7067	4.05450	2.34086	-4.3653	15.7786	1.28	9.24
Total	132	3.2017	3.24680	.28260	2.6426	3.7607	.64	18.59

No significant differences were detected in schneiderian membrane thickness between male and female subjects. ANOVA did not show any statistically significant differences in the frequencies of endodontic‒periodontal lesions and an increase in schneiderian membrane thickness. Furthermore, intra-group evaluations with Tukey test showed no significant differences between the different groups with endodontic‒periodontal lesions.

Pearson’s correlation coefficient showed significant relationships between periapical and periodontal infections (P<0.001), palate depth (P=0.019) and schneiderian membrane thickness. There were no significant relationships between periapical and other variables. In addition, there were significant relationships between periodontal infection and the depth of the palate (P<0.001), the distance between the sinus floor and the root apices (P=0.03), and the thickness of the schneiderian membrane (P=0.37). In addition, there were significant relationships between the height of the crest and the depth of the palate (P=0.016), the distance from the sinus floor to the root apices (P<0.001) and schneiderian membrane thickness (P=0.042). Furthermore, there was a significant relationship between the thickness of the schneiderian membrane and the distance from the sinus floor to the root apices (P=0.38) (Table 2).

**Table 2:** Correlation between different variables

		p.a.inf	perio.inf	pal. depth	BP. width	os. height. apex	os. height. crest	schneiderian
p.a.inf	Pearson Correlation	1	.369^**^	-.0181^*^	.100	.120	.072	.356^**^
	Sig. (1-tailed)		.000	.019	.126	.085	.206	.000
	N	132	132	132	132	132	132	132
perio.inf	Pearson Correlation	.369^**^	1	-.303^**^	-.019	.164^*^	.045	.156^*^
	Sig. (1-tailed)	.000		.000	.416	.030	.306	.037
	N	132	132	132	132	132	132	132
pal. depth	Pearson Correlation	-.181^*^	-.303^**^	1	.061	.094	.187^*^	-.074
	Sig. (1-tailed)	.019	.000		.242	.142	.016	.198
	N	132	132	132	132	132	132	132
BP. width	Pearson Correlation	.100	-.019	.061	1	.139	.095	-.111
	Sig. (1-tailed)	.126	.416	.242		.056	.138	.103
	N	132	132	132	132	132	132	132
os. height. apex	Pearson Correlation	.120	.164^*^	.094	.139	1	.728^**^	-.155^*^
	Sig. (1-tailed)	.085	.030	.142	.056		.000	.038
	N	132	132	132	132	132	132	132
os. height. crest	Pearson Correlation	.072	.045	.187^*^	.095	.728^**^	1	-.151^*^
	Sig. (1-tailed)	.206	.306	.016	.138	.000		.042
	N	132	132	132	132	132	132	132
schneiderian	Pearson Correlation	.356^**^	.156^*^	-.074	-.111	-.155^*^	-.151^*^	1
	Sig. (1-tailed)	.000	.037	.198	.103	.038	.042	
	N	132	132	132	132	132	132	132

** Correlation is significant at the 0.01 level (1-tailed). * Correlation is significant at the 0.05 level (1-tailed).

The results of regression analysis showed that of all the variations evaluated, there was a significant relationship between periapical infection and schneiderian membrane thickness.

## Discussion

Detailed information is necessary about the maxillary sinus anatomy and its anatomic variations for safe surgeries in the maxillary posterior area. Proper knowledge about the anatomy of the area involved results in precise surgery and prevents complications.^26^ Several studies have investigated maxillary sinus septa, concluding that these septa are more common in edentulous patients with atrophic maxilla than those with dentate maxilla, with the septa in edentulous atrophic maxilla being usually shorter than those in dentate maxilla. Additionally, the prevalence rate of septa has not been correlated with patient age and sex.^26-29^ Many authors have considered the presence of septa if the height is estimated >2.5 mm.^28,30^ In a study by Lee et al, the location of septa was investigated, and a higher prevalence rate was reported in the middle region (from the distal aspect of the second premolar to the distal aspect of the second molar) (50%), followed, in descending order, by the anterior region (mesial to the distal aspect of the second premolar) (24.0%) and the posterior region (the distal aspect of the second molar region) (22.7%),^31^ consistent with the results of previous studies.^32^

Conventional radiographic techniques are used to diagnose an increase in the thickness of maxillary sinus membrane and apical periodontitis; these techniques include conventional radiography (x-ray), MRI, CT and conventional periapical radiography. CBCT is a novel technique and has been used in recent years in oral surgeries, orthodontic evaluations, implant treatment planning, evaluation of apical periodontitis and periodontal treatment planning.^33^ Some studies have reported that the results of evaluation of hard tissues with the CBCT technique are comparable to those carried out with the use of CT and conventional radiographic techniques.^34-37^ CBCT images are effective in revealing the etiology and relationship between odontogenic pathologic lesions and sinus involvement.^38^

CBCT technique was used in this study to evaluate the demographic and radiographic characteristics of the schneiderian membrane and the relationship between changes in schneiderian membrane thickness and tooth pathologic lesions such as periapical and periodontal infections, endodontic‒periodontal involvement and some anatomic features of areas adjacent to the maxillary sinus such as the depth of the palate and the thickness of bone between the sinus cavity floor and root apices of molar teeth and the alveolar crest. In order to achieve high-resolution images, FOV was confined to the upper jaw.

Vallo and Aimetti considered the thickness of the gingiva and gender as reliable genetic parameters for estimation of schneiderian membrane thickness, reporting that it was thicker in subjects with thick gingival biotype and thinner in females;^39,40^ however, in the present study, there was no significant difference in the thickness of the schneiderian membrane between males and females. In addition, it was shown that there was no relationship between the thickness of the schneiderian membrane and endodontic‒periodontal lesions in the maxillary posterior teeth. Eggmann et al showed, in a systematic review, an association between periapical lesions in the posterior maxilla and schneiderian membrane thickness, with no significant relationship between periodontal diseases and schneiderian membrane thickness, indicating contradictory results.^41^

In the present study, the presence of periapical infection resulted in an increase in schneiderian membrane thickness, consistent with the results of a study by Yulu et al, in which the presence of periapical infection resulted in an increase in the thickness of the mucosa in 48.4% of the cases.^8^ In comparison, this percentage was reported to be 38.1% in a study by Ritter et al,^42^ 83.2% in a study by Belger et al^43^ and 60% in a study by Hahnel et al.^44^ Scanning based on MRI technique showed a prevalence rate of 50%.^45^ The discrepancies between the results of studies might be attributed to differences in race or age and the different diagnostic techniques used.

Furthermore, in the current study, a significant relationship was detected between periodontal infection and an increase in schneiderian membrane thickness. In a study by Brullmann et al,^17^ CBCT evaluations showed significant relationships between schneiderian membrane thickness and the presence of carious teeth in the posterior maxilla and periodontitis. However, Dagassan-Bernd tet al^46^ were unable to use clinical periodontal parameters for the estimation of schneiderian membrane thickness.

In the present study, a significant relationship was detected between the location of maxillary posterior teeth, i.e. the thicken of bone between the apex and the maxillary sinus floor and also between the crest of the alveolar bone and the maxillary sinus floor, and the thickness of the schneiderian membrane, consistent with the results of a study by Janner et al.^3^ Dagassan-Berndt, also, reported a strong relationship between the height of the bone above the root apex in the molar area and schneiderian membrane thickness.^46^

One of the limitations of the present study was inclusion of patients that had maxillary sinusitis in association with thickening of the mucosa; however, not all the patients with maxillary sinusitis exhibit a thickening of the membrane and mucosa. In addition, in the present study, it was not possible to access the patients and evaluate their clinical conditions. Also, the edentulous areas were not evaluated since such regions did not have a proper reference point such as a maxillary first molar tooth.

## Conclusion

Under the limitations of the present study, there was an association between the presence of periapical lesions and periodontal infections in the posterior area of the maxilla and thickening of the schneiderian membrane. In addition, there was a significant relationship between the location of maxillary posterior teeth, i.e. the thickness of bone from the root apex to the maxillary sinus floor and also from the alveolar crest to the maxillary sinus floor, and schneiderian membrane thickness.

## Acknowledgment

The authors would like to thank the Kaviani & Johari Oral and Maxillofacial Radiology Cener for their cooperation with this study.

## Authors’ contributions

AK prepare proposals, set and enter the results of the studies and their interpretation, Prepare and interpret data, Prepare a final report, prepare results, writing the article AS supervised the design and execution of the study and Preparing a final report. AS collected the data and contributed to preparation of the proposal.

## Funding

No funding was requested for this study.

## Competing interests

The authors declare no competing interests with regards to the authorship and/or publication of this article.

## Ethics approval

The ethics approval for this study was obtained from the ethics committee of Tabriz University of Medical Sciences.
